# Hepatic Models in Precision Medicine: An African Perspective on Pharmacovigilance

**DOI:** 10.3389/fgene.2022.864725

**Published:** 2022-04-14

**Authors:** Tracey Hurrell, Jerolen Naidoo, Janine Scholefield

**Affiliations:** ^1^ Bioengineering and Integrated Genomics Group, Next Generation Health Cluster, Council for Scientific and Industrial Research, Pretoria, South Africa; ^2^ Department of Human Biology, Faculty of Health Sciences, University of Cape Town, Cape Town, South Africa

**Keywords:** pharmacovigilance, iPSCs, hepatocyte, African precision medicine, ADRs

## Abstract

Pharmaceuticals are indispensable to healthcare as the burgeoning global population is challenged by diseases. The African continent harbors unparalleled genetic diversity, yet remains largely underrepresented in pharmaceutical research and development, which has serious implications for pharmaceuticals approved for use within the African population. Adverse drug reactions (ADRs) are often underpinned by unique variations in genes encoding the enzymes responsible for their uptake, metabolism, and clearance. As an example, individuals of African descent (14–34%) harbor an exclusive genetic variant in the gene encoding a liver metabolizing enzyme (CYP2D6) which reduces the efficacy of the breast cancer chemotherapeutic Tamoxifen. However, CYP2D6 genotyping is not required prior to dispensing Tamoxifen in sub-Saharan Africa. Pharmacogenomics is fundamental to precision medicine and the absence of its implementation suggests that Africa has, to date, been largely excluded from the global narrative around stratified healthcare. Models which could address this need, include primary human hepatocytes, immortalized hepatic cell lines, and induced pluripotent stem cell (iPSC) derived hepatocyte-like cells. Of these, iPSCs, are promising as a functional *in vitro* model for the empirical evaluation of drug metabolism. The scale with which pharmaceutically relevant African genetic variants can be stratified, the expediency with which these platforms can be established, and their subsequent sustainability suggest that they will have an important role to play in the democratization of stratified healthcare in Africa. Here we discuss the requirement for African hepatic models, and their implications for the future of pharmacovigilance on the African continent.

## Genetic Diversity in Africa and its Impact on Healthcare

The global population is becoming increasingly reliant on pharmaceuticals as healthcare systems are burdened by lifestyle diseases, drug resistance, mental health issues, and innumerable orphan diseases. While substantial progress has been made to ensure patients in Africa have access to treatments, the majority of pharmaceuticals are trialed on narrow genetic populations using both preclinical models and population cohorts that are not inclusive of global, especially African, diversity. This is well exemplified by the fact that the African continent currently contributes only ∼3.3% of the estimated 393 000 active clinical trials globally (ClinicalTrials.gov, 2021). Yet, despite its disproportionately high disease burden, Africa tolerates suboptimal treatment regimens which affect a significant portion of the continent (Mpye et al., 2017).

It has long been understood that genetic variants in drug metabolizing genes are a major factor resulting in altered drug responses (Weinshilboum, 2003; Schärfe et al., 2017). Consequently, the extraordinary genetic diversity in the African population, compounded by the underrepresentation of this diversity in preclinical development, is likely to be driving a high prevalence of severe adverse drug reactions (ADRs) within the populus (Rajman et al., 2017). This may in turn decrease adherence to treatment regimens, often in vulnerable population groups. Understanding the magnitude of this issue is further complicated by the likely under-reporting of African ADRs on Vigibase - the global Individual Case Safety Report database (Ampadu et al., 2016). Despite increasing inclusion in studies/resources such as the Genome Aggregation Database (gnomAD), the 1000 Genomes Project, the African Genome Variation Project (AGVP), and the H3Africa project (Gurdasani et al., 2015), the clinical impact of Africa’s genetic diversity on ADRs is only now coming to the forefront (da Rocha, 2021). While still striving to address the inequality in the global representation of African genomes (Wonkam, 2021), how then do we best serve the people of Africa, and successfully implement precision medicine on the continent?

In the context of ADRs, precision medicine aims to maximize the probability of clinical success, by matching patients with predicted best treatment options, either for individuals (personalized medicine) or well-defined subsets of the population (stratified medicine). The mitigation of ADRs through regulatory interventions represents a valuable and tenable strategy for the implementation of stratified medicine within Africa, especially for drugs that are highly utilized across the continent. These regulatory changes must first be driven by rigorous scientific efforts to bridge the disconnect that exists between the identification of African relevant genetic variants, and the functional validation of their potential clinical impact. Here we outline why we believe cellular hepatic models would be a useful tool to validate African relevant gene-drug interactions.

## The Role of the Liver in Pharmacovigilance

Hepatocytes, accounting for 60–70% of the total liver cell population, play a central role in the biotransformation, intermediary and energetic metabolism of endogenous and exogenous xenobiotics, including pharmaceuticals (LeCluyse et al., 2012). Hepatic metabolism of xenobiotics to more polar and hydrophilic counterparts facilitate their excretion and prevents toxic accumulation. This specialized detoxification machinery relies on the selectivity, abundance, expression, and interplay of sequentially coordinated (Phase 0, I, II and III) metabolizing enzymes (Zhang and Surapaneni, 2012). The CYP450 family, is a membrane-associated, hydrophobic enzyme system, functionally intended to be cyto-protective. However, CYP450 enzymes are also responsible for forming pharmaceutically active metabolites of prodrugs, and can generate toxic reactive intermediary products (Liebler and Guengerich, 2005).

CYP450 enzymes are of relevance to pharmaceuticals as their induction/inhibition potential, genetic polymorphisms, epigenetic regulation and non-genetic host factors such as gender, age, disease(s), and polypharmacy can contribute to functional disparities. CYP450 enzymes are responsible for approximately 80% of hepatic Phase I metabolism, with 12 isoforms being responsible for ∼75% of Phase I oxidation reactions (Evans and Relling, 1999). In a literature survey for drug metabolism pathways with known CYP450 involvement (248 drugs), the fraction of clinically used drugs metabolized by CYP450 isoforms approximates the following: 3A4/5: 30.2%, 2D6: 20%, 2C9: 12.8%, 1A2: 8.9%, 2B6: 7.2%, and 2C19: 6.8%. However enzyme functionality does not correlate linearly with hepatic enzyme expression, for example, CYP2D6 accounts for 1.3–4.3% of the hepatic pool and metabolizes 20% of clinically used drugs, whereas CYP2B6 accounts for 1.7–5.3% while metabolizing ∼7% (Zanger and Schwab, 2013).

The functional consequence of genetic polymorphisms leads to the classification of pharmacokinetic phenotypes as poor, intermediate, extensive/normal, and ultra-rapid metabolizers. Thus, contextualizing major genetic determinants of drug metabolism in different populations is essential to the provision of safe and efficacious pharmaceuticals (Belle and Singh, 2008). Investment in, and implementation of, national genomic-medicine initiatives is driving transformation in healthcare (Stark et al., 2019). However, the adoption of pharmacogenetics testing in sub-Saharan Africa (SSA) faces numerous clinical, scientific, technical, socio-economic, and governance barriers (Tata et al., 2020).

## Clinical Implications and Challenges of Genotyping Pharmacogenes

Given the genetic diversity in Africa, executing precision medicine strategies could be considered beyond the capabilities of the healthcare infrastructure, in the predominantly developing nations which constitute the region. Yet, there are well defined examples of gene-drug pairs where ADRs could be minimized or resolved thereby significantly alleviating socio-economic impact.

One example of such a gene-drug pair, for a non-communicable disease, is that of the breast cancer drug Tamoxifen and its key liver metabolizing gene. Tamoxifen is a selective estrogen receptor modulator, which relies on CYP2D6 to catalyze the formation of primary and secondary metabolites which have higher anti-estrogenic activity. The CYP2D6 gene locus is highly polymorphic, with the allelic variant CYP2D6*17 prevalent in 21.7% of the African population [14–34% in African sub-cohorts (Nemaura et al., 2012; Masimirembwa and Hasler, 2013)]; yet it is nearly absent in Europeans, Asian, and admix American populations. African individuals harboring CYP2D6*17 have decreased enzyme expression and activity, resulting in reduced efficacy and increased ADRs, consequently promoting the risk of cancer recurrence and diminishing the likelihood of positive clinical outcomes (Higgins and Stearns, 2010).

Another concerning gene-drug pair is that of CYP2B6 and the antiretroviral Efavirenz. CYP2B6 variants, with a higher prevalence in the African population, include functionally deficient haplotypes CYP2B6*6 and CYP2B6*18 (Langmia, 2021). In comparison to European populations, even a small increase in the prevalence of these variants in the context of SSA has a significant impact. These poor metabolizers when administered Efavirenz, which is used as first-line antiretroviral therapy for adults in South Africa, have elevated plasma concentrations and decreased clearance which can precipitate severe neurotoxicities including catatonia, suicidal ideation, and psychosis (Masimirembwa and Hasler, 2013). With the prevalence of HIV in European and SSA populations at 5 and 25% respectively, it is easy to see the positive impact that a pharmacovigilance-based precision medicine strategy would have on the disease burden in SSA.

Here we outlined two of the many well-established gene-drug pairs within the African context. Yet, data from the 1,000 Genomes Project indicates that SSA contains 25% more genetic diversity than the rest of the world (1000 Genomes Project Consortium 2015) rendering the absolute requirement for studies which validate the relationship(s) between host genetic and pharmaceutical interactions. Furthermore, since genes such as CYP2D6 and CYP2B6 are responsible for the metabolism of ∼27% of clinically approved drugs (Zanger and Schwab, 2013), it begs the question, how can we model the contribution of these and other genetic variants to ADRs and in doing so, provide best treatment outcomes.

The African Pharmacogenomics Consortium (APC) was launched in 2018, with the mandate to educate, build capacity, capability, governance, and technologies to promote the use of pharmacogenomics for the clinical benefit of African patients (Dandara, 2019). However, pharmacogenetic screening using commercial genotyping applications have historically been ineffective due to variants which are exclusive to the African population not being adequately represented (Dodgen et al., 2013). Driving impact into the clinical space, by the APC and other such initiatives, will require sustained contributions from stakeholders and investment in local population-relevant biotechnology. Given that pharmacogenomics is fundamental to precision medicine, and that screening for genetic variants is not yet considered a point-of-care prior to dispensing drugs (Tata et al., 2020), Africa needs to invest further in technologies which directly contribute to stratified healthcare for its people. With clinical trials incurring the largest financial expenditure of the drug development pipeline, technologies which provide an opportunity to screen drugs within a genetically and physiologically relevant background must be established.

## Hepatocyte Models in Precision Medicine

Safety pharmacology informs risk-benefit relationships by assessing adverse effect liability and safety margins. Hepatotoxicity and aberrant xenobiotic metabolism are major contributors to post-marketing drug withdrawal, failure of investigational new drugs, and drug inefficacy. Population risk-benefit ratios, derived from clinical trial data, inform regulatory decisions despite knowing that patient-level responses will differ in terms of efficacy and adverse outcome risk. Importantly, as the contribution of an individual’s genetics to the risk-benefit relationship is better understood it is possible that reducing attrition rates during development, using the current testing paradigms, will not be synonymous with global improvement in patient outcomes (Ahuja and Sharma, 2014; Dambach et al., 2016; Atienzar and Nicolas, 2018; Babai et al., 2018).

Predicting treatment inefficacy and ADRs is becoming more challenging as our understanding of the impact which inter-ethnic and inter-individual genetics has on healthcare improves. Establishing effective strategies to validate the impact of Africa’s unique population genetics on clinical outcomes is imperative for improved pharmacovigilance, and the successful implementation of precision medicine on the African continent. While numerous preclinical models of xenobiotic metabolism exist, those with the potential to validate the impact of individual, African-relevant genetic variants on xenobiotic metabolism are lacking. Preclinical hepatocyte and liver models include: 1) primary human hepatocytes (PHHs); 2) subcellular fractions including microsomes or S9 fractions; 3) cell lines; 4) *ex vivo* tissue slices; 5) isolated perfused organs; 6) genetically engineered cells expressing metabolizing enzymes; 7) cell-free and in silico approaches; and 8) animal models (Zeilinger et al., 2016; Yamasaki et al., 2020). However, ethical, economic and practical considerations dictate the choice of model, with trade-offs between functional complexity, applicability, cost, scalability, expedience, and accessibility (Costa et al., 2014). The focus here will be on the potential use case for *in vitro* cell-based models of xenobiotic metabolism.

### Primary Human Hepatocytes

PHHs are the “gold standard” or “historical standard” for modeling xenobiotic metabolism (LeCluyse et al., 2012). PHHs express all major Phase I and Phase II drug-metabolizing enzymes and transporter proteins at functional levels, with their value being exemplified by their application as models of drug-induced liver injury (Bell et al., 2016), liver metabolism (Vorrink et al., 2017), and liver diseases; including cholestasis (Hendriks et al., 2016), steatosis (Kozyra et al., 2018; Prill et al., 2019) and fibrosis (Pingitore et al., 2019; Hurrell et al., 2020). In this respect, PHHs have a clear advantage over other cellular models in terms of the fidelity with which they can recapitulate liver physiology and function (Ingelman-Sundberg and Lauschke, 2022).

PHHs are however prone to rapidly declining metabolic function and dedifferentiate, within hours, in traditional monolayer cultures (Lauschke et al., 2016; Heslop et al., 2017). The adoption of 3-dimensional (3D) culture methods has largely overcome this limitation, improving the functional longevity of PHHs in culture (>21 days) through enhanced mimicry of endogenous architecture and physiological cues. This has been achieved using 3D spheroid models (Bell et al., 2016; Vorrink et al., 2017), microfluidic liver-on-a-chip systems (Beckwitt et al., 2018), microscale bioreactors (Freyer et al., 2018), and 3D bioprinted livers (Otieno et al., 2018).

However, donor age, genetics and pathophysiology, as well as the impact of cryopreservation on attachment efficiency/viability and consequently their functional properties, are important factors to consider when using PHHs (Ölander et al., 2019). Furthermore, the inability of PHHs to self-renew and their inaccessibility to the broader research community, present significant challenges to the scalability and use of PHH models, especially for research applications which require population-level interrogation of biological phenotypes (Otieno et al., 2018; Lauschke et al., 2019).

### Immortalized Hepatic Cell Lines

Unlike PHHs, immortalized hepatic cell lines harbor a potentially unlimited capacity for self-renewal. Numerous cell lines have been used to model liver and its associated function and pathologies including; human hepatocellular carcinomas (HCC) such as HepG2 and HepB3, Fa2N4 cells, bipotent hepatic progenitors (HepaRG), and HepG2 clonal variants, HepG2/C3A (Qiu et al., 2015). Due to the ease of use and broad accessibility, HepG2 cells are among the most commonly used *in vitro* hepatocyte model despite exhibiting reduced basal gene expression of Phase I and Phase II drug-metabolizing enzymes, as well as functional and phenotypic responses that are inconsistent with those of PHHs (LeCluyse et al., 2012; Zhou and Fan, 2019).

HepaRG cells can be differentiated to express numerous metabolizing enzymes and transporters at levels that are superior to those of other carcinoma lines (Szabo et al., 2013; Mayati et al., 2018). They are functionally stable for weeks following differentiation and like other HCCs have also been used as a surrogate for PHHs to model various liver pathologies, hepatotoxicity and xenobiotic metabolism (Andersson et al., 2012; Mayati et al., 2018). However, the propensity of oncogenic lines to cumulatively acquire genetic perturbations and chromosomal rearrangements (e.g. HepG2s show distinct aneuploidy; Wong, 2000) presents a challenge in their use, particularly in the extrapolation of clinically relevant assertions (Zhou and Fan, 2019). These factors can significantly impact the underlying biology and impartiality of these models in addressing specific questions, for example, assessing the contribution of genetic variance to functional phenotypes.

The generation of immortalized PHH lines, with the capacity for self-renewal, is an interesting prospect for future research efforts as various immortalization strategies and use cases have been described (Ramboer et al., 2014, 2015; Collins et al., 2020). These lines however share some of the disadvantages of their carcinoma-derived counterparts in terms of diminished and (or) limited functionality in comparison to PHHs, along with the negative impact of sustained proliferative cues on genomic stability (Ramboer et al., 2014, 2015). The generation of immortalized lines that overexpress specific CYP450 enzymes and hepatocyte specification factors, alongside conditional immortalization strategies, have also been explored as compensatory mechanisms to enhance the robustness of these models (Ramboer et al., 2015).

### Induced Pluripotent Stem Cell Derived Hepatocyte Models

Reprogramming of somatic cells to induced pluripotent stem cells (iPSCs) is achieved by the stochastic overexpression of key stem cell transcription factors (Takahashi et al., 2007). Similar to differentiation protocols for embryonic stem cells, hepatocyte differentiation from iPSCs is mimicked using a multistage cascade via endoderm, anterior definitive endoderm, and hepatocyte commitment through to hepatocyte-like cells (HLCs) (Si-Tayeb et al., 2010; Touboul et al., 2010; Hannan et al., 2013; Mathapati et al., 2016). Failure to express drug metabolizing enzymes, at levels comparable to PHH (Baxter et al., 2015; Sampaziotis et al., 2015) is being improved as the complex mimicry of liver development is better recapitulated by differentiation protocols which direct cellular fate with greater fidelity (Ouchi et al., 2019; Raggi et al., 2022; Takeishi et al., 2020). In addition to these more physiologically relevant models, the improved benchmarking of liver metabolizing enzymes compared to both fetal and adult counterparts (Zabulica et al., 2019), is better defining how HLCs can be appropriately applied to biological questions.

Similar to PHHs, iPSCs retain the original genetic complement of the individual from which they were derived, but with the added advantage of self-renewal. This characteristic further confers genome engineering capabilities to iPSCs (Hockemeyer and Jaenisch, 2016; Sanjurjo-Soriano, 2021). Consequently, they represent a confluence of enabling methodologies in a single model i.e. an infinite source of cellular material representing an individual genome amenable to gene editing and capable of lineage specific differentiation to all three germ layers.

While immortalized cells are also amenable to genome engineering, the improved chromosomal stability and physiological relevance of iPSC derived HLCs lends a significant advantage relevant to the African context. Human iPSC-HLC models have been used to model the disruption of the ER in α1-antitrypsin deficiency (Yusa et al., 2011; Segeritz et al., 2018), and urea cycle defects (Zabulica et al., 2021) by correcting patient-specific cells using ZFN and CRISPR/Cas technologies respectively, to erase the disease signature. The success of these bioengineered models, along with the functional activity of iPSC-HLCs being improved by scalable 3D technologies such as spheroids (Takebe et al., 2017; Heidariyan et al., 2018; Rashidi et al., 2018) and liver-on-a-chip approaches (Kamei et al., 2019), is diversifying their applications in basic and translational research.

### How do These Models Interplay and What is Their Value in Pharmacovigilance

In recent years, it has been increasingly recognized that improving preclinical predictions is dependent on the use of relevant cellular material, modeling native architecture, genetic background, and the stringency of defining a model’s suitability to specific functional applications. Disparities exist in access to *in vitro* models from diverse genetic backgrounds, as donor demographics of PHHs are overwhelmingly in favor of Caucasian populations ((2 Caucasian, 1 other (Vorrink et al., 2018); 45 Caucasian, 1 other (Baze et al., 2018); 8 Caucasian (Hurrell et al., 2020)). Similarly, of the 2,912 globally registered iPSC lines in the Human Pluripotent Stem Cell Registry (HPSC registry, 2021) only 35 lines from 18 individuals are recorded to have potential African ancestry (annotated as black/African-American/mixed ethnicity), and both HepG2 and HepaRG cell lines were derived from individuals of Caucasian descendant. This highlights the lack of genetic diversity within the laboratory models and remains one of the reasons that bias is still perpetuated in current scientific literature. These circumstances highlight the need for the expansion of an *in vitro* repertoire of “African” liver models (Moore et al., 2021).

Bioengineered iPSC-HLCs derived from individuals of African ancestry, could provide an ideal platform to empirically assess the relationship between individual and cumulative African specific genetic variants and pharmacokinetic phenotypes within an isogenic background. While PHHs have an invaluable role to play in assessing inter-individual variation, and the validation of pharmacokinetic phenotypes, population stratification metrics need to be more stringently and singularly assessed. PHHs may remain the “gold standard” for determining pharmacokinetic phenotypes, however, access to the number of clinically relevant PHHs required to mirror the diversity in Africa is currently not feasible in terms of scalability, accessibility, and cost. Likewise, while the use of immortalized and carcinoma derived hepatic cell lines may present specific challenges for modeling pharmacokinetically relevant genotypes; the derivation of such lines from individuals of African ancestry would facilitate a readily accessible way for researchers to reshape the global underrepresentation of African genetics in science and improve the transferability of global search findings to an African relevant context. The necessity for these resources is perhaps most easily exemplified by the assessment of a few of our own iPSC lines where we focus on generating lines from donors of African origin. Here we identified a line which is both a poor metabolizer for CYP2B6 (CYP2B6*6/*18) as well as a reduced metabolizer for CYP2D6 (CYP2D6*1/*17), leading to almost complete ablation of Efavirenz metabolism and aberrant production of Tamoxifen’s potent bioactive metabolites respectively. This is not remarkable within the context of the prevalence in Africa but would be exceptionally rare globally as iPSC lines are predominantly derived from Caucasians.

## Addressing Precision Medicine Challenges Faced by Africa

New scientific breakthroughs, and improved access to these technologies on the African continent, has positioned scientists to establish platforms which directly impact the burden of clinical healthcare. Access to enabling technologies including; genome engineering, iPSC differentiation, and malleable 3D biomimetic microenvironments, provides synthetic biology with tools to develop preclinical models with improved capacity for predictive extrapolation. While individualized treatments might remain an unrealistic goal in Africa, comprehensive profiling of genetic variants which impact pharmacokinetic/dynamic profiles would aid in the implementation of subpopulation stratification and subsequently improve healthcare strategies on the African continent. With the proposed landscape, independent, interdependent or sequentially applied models could be used to provide empirical evidence for the efficacy or inefficacy of marketed/prescribed drugs in clinical practice.

**FIGURE 1:**
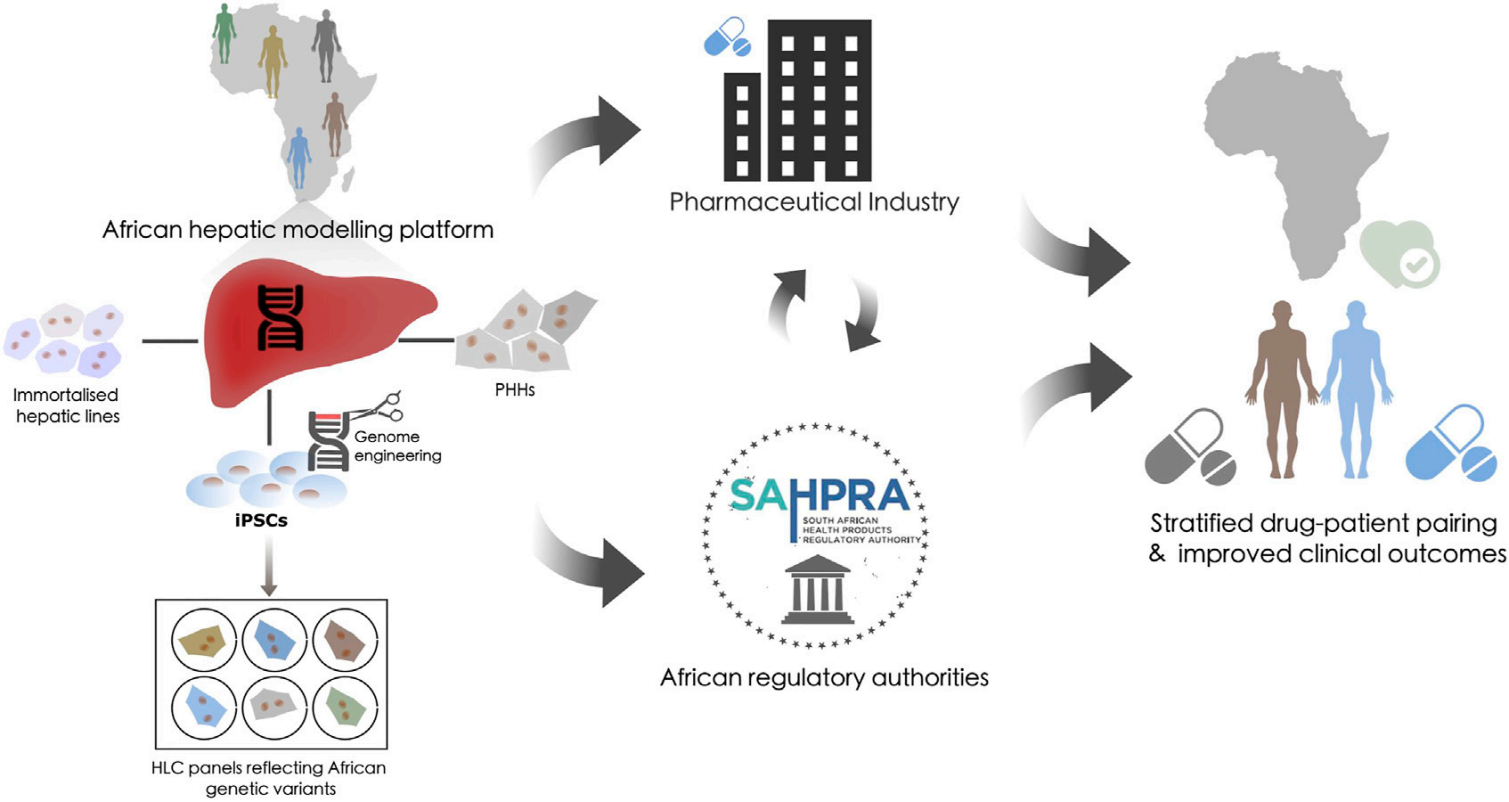
The potential role of a hepatic modeling platform in guiding pharmacovigilance. Proposal for an integrative *in vitro* African pharmacovigilance platform that houses various hepatic models. Such a modeling platform would utilize immortalized hepatic lines, primary human hepatocytes (PHH), and induced pluripotent stem cells (iPSCs) from donors of African origin. iPSC could be genome engineered to generate panels of hepatocyte-like cells (HLCs) to specifically validate African-relevant genetic variants against efficacy/ADRs within an isogenic background. This platform would allow these models to be applied independently, interdependently, or sequentially to recapitulate and validate the xenobiotic metabolism of the liver across the genetic diversity of the African continent. This could then be used to inform decision-making in the global pharmaceutical industry and within the African regulatory landscape to stratify drug-patient pairing and improve clinical outcomes.

To address these challenges, we propose an integrative *in vitro* pharmacovigilance platform that houses various hepatic models that recapitulate the xenobiotic metabolism of the liver to specifically link African-relevant genetic variants to efficacy/ADRs. These tools would support pharmacogenetic based decision-making and reduce the use of inadequate or harmful pharmaceutical interventions (Figure 1). While the requirement for pharmacogenetic-based treatment stratification has been clinically evidenced in African populations for over a decade (Ngaimisi et al., 2011; Shrif et al., 2011; Masimirembwa and Hasler, 2012; van der Merwe et al., 2012), few national genomic-medicine initiatives/studies, or national regulatory guidelines exist (Radouani et al., 2020) which ingrain pharmacogenetics into the African clinical landscape. The consequence of which is that for some populations we will continue to fail in pairing the right drug, at the right dose, with the right patient to address their healthcare needs. Successful utilization of a platform that can address xenobiotic metabolism from multiple vantage points could lead to intervention in multiple avenues: 1) providing definitive diagnostic assays from proven genetic variant/drug relationships, 2) stratification of drug-patient pairing with improved efficacy outcomes, and 3) providing a model to evaluate redesigned pharmaceutical compounds.

Our research group, which successfully derived one of the first human iPSC lines on the African continent, has applied genome engineering strategies to edit iPSCs, and has been actively engaged in modeling iPSC-HLCs and other “disease-in-a-dish” models. Further to this, isolating PHHs from individuals of African descent, on the continent itself, would represent a significant milestone for the accessibility and utilization of genetically representative PHHs within the African research landscape. However, this will require key stakeholders from multidisciplinary backgrounds to collectively establish such an Afrocentric resource within the global pharmacovigilance arena. Solidifying the foundation of an African hepatic modeling platform will require concerted efforts to establish a new paradigm for preclinical and clinical research collaborations that collectively drive impact in healthcare. The convergence of a number of these traditionally siloed technologies and methodologies, within an established African research infrastructure, would support the feasibility of a hepatic modeling platform and serve as a launchpad for national and ultimately continent-wide initiatives.

## Data Availability

The original contributions presented in the study are included in the article/Supplementary Material, further inquiries can be directed to the corresponding author.

## References

[B1] Ahuja V. Sharma S. (2014). Drug Safety Testing Paradigm, Current Progress and Future Challenges: an Overview. J. Appl. Toxicol. 34 (6), 576–594. 10.1002/jat.2935 24777877

[B2] Ampadu H. H. Hoekman J. de Bruin M. L. Pal S. N. Olsson S. Sartori D. (2016) Adverse Drug Reaction Reporting in Africa and a Comparison of Individual Case Safety Report Characteristics between Africa and the Rest of the World: Analyses of Spontaneous Reports in VigiBase®, Drug Saf., 39. 335–345. 10.1007/s40264-015-0387-4 26754924 PMC4796322

[B3] Andersson T. B. Kanebratt K. P. Kenna J. G. (2012). The HepaRG Cell Line: a Uniquein Vitrotool for Understanding Drug Metabolism and Toxicology in Human. Expert Opin. Drug Metab. Toxicol. 8 (7), 909–920. 10.1517/17425255.2012.685159 22568886

[B4] Atienzar F. A. Nicolas J.-M. (2018). “Prediction of Human Liver Toxicity Using *In Vitro* Assays: Limitations and Opportunities,” in Drug-Induced Liver Toxicity. Editors Chen M. Will Y. (New York, NY: Springer Methods in Pharmacology and Toxicology), 125–150. 10.1007/978-1-4939-7677-5_7

[B5] Babai S. Auclert L. Le-Louët H. (2021). Safety Data and Withdrawal of Hepatotoxic Drugs. Therapies 76, 715–723. 10.1016/j.therap.2018.02.004 29609830

[B6] Baxter M. Withey S. Harrison S. Segeritz C.-P. Zhang F. Atkinson-Dell R. (2015). Phenotypic and Functional Analyses Show Stem Cell-Derived Hepatocyte-like Cells Better Mimic Fetal rather Than Adult Hepatocytes. J. Hepatol. 62 (3), 581–589. 10.1016/j.jhep.2014.10.016 25457200 PMC4334496

[B7] Baze A. Parmentier C. Hendriks D. F. G. Hurrell T. Heyd B. Bachellier P. (2018). Three-Dimensional Spheroid Primary Human Hepatocytes in Monoculture and Coculture with Nonparenchymal Cells. Tissue Eng. C: Methods 24 (9), 534–545. 10.1089/ten.tec.2018.0134 30101670

[B8] Beckwitt C. H. Clark A. M. Wheeler S. Taylor D. L. Stolz D. B. Griffith L. (2018). Liver 'organ on a Chip'. Exp. Cel Res. 363 (1), 15–25. 10.1016/j.yexcr.2017.12.023 PMC5944300 29291400

[B9] Bell C. C. Hendriks D. F. G. Moro S. M. L. Ellis E. Walsh J. Renblom A. (2016). Characterization of Primary Human Hepatocyte Spheroids as a Model System for Drug-Induced Liver Injury, Liver Function and Disease. Sci. Rep. 6 (1), 25187. 10.1038/srep25187 27143246 PMC4855186

[B10] Belle D. J. Singh H. (2008). Genetic Factors in Drug Metabolism. Am. Fam. Physician 77 (11), 1553–1560. 18581835

[B11] B. Tata E. A. Ambele M. Pepper S. (2020). Barriers to Implementing Clinical Pharmacogenetics Testing in Sub-saharan Africa. A Critical Review. Pharmaceutics 12 (9), 809. 10.3390/pharmaceutics12090809 PMC7560181 32858798

[B12] ClinicalTrials.gov (2021). clinicaltrials.gov. Available at: https://clinicaltrials.gov/ct2/search/map (Accessed October 29, 2021).

[B13] Collins D. P. Hapke J. H. Aravalli R. N. Steer C. J. (2020). Development of Immortalized Human Hepatocyte-like Hybrid Cells by Fusion of Multi-Lineage Progenitor Cells with Primary Hepatocytes. PLOS ONE 15 (6), e0234002. 10.1371/journal.pone.0234002 32497071 PMC7272032

[B14] Costa A. Sarmento B. Seabra V. (2014). An Evaluation of the Latestin Vitrotools for Drug Metabolism Studies. Expert Opin. Drug Metab. Toxicol. 10 (1), 103–119. 10.1517/17425255.2014.857402 24205859

[B15] da Rocha J. E. B. (2021). The Extent and Impact of Variation in ADME Genes in Sub-saharan African Populations. Front. Pharmacol. 12. Available at: https://www.frontiersin.org/article/10.3389/fphar.2021.634016 (Accessed: 21 January 2022). 10.3389/fgene.2021.626954 PMC8549571 34721006

[B16] Dambach D. M. Misner D. Brock M. Fullerton A. Proctor W. Maher J. (2016). Safety Lead Optimization and Candidate Identification: Integrating New Technologies into Decision-Making. Chem. Res. Toxicol. 29 (4), 452–472. 10.1021/acs.chemrestox.5b00396 26625186

[B17] Dandara C. (2019). African Pharmacogenomics Consortium: Consolidating Pharmacogenomics Knowledge, Capacity Development and Translation in Africa. AAS Open Res. 10.12688/aasopenres.12965.1 PMC7194139 32382701

[B18] Dodgen T. M. Hochfeld W. E. Fickl H. Asfaha S. M. Durandt C. Rheeder P. (2013). Introduction of the AmpliChip CYP450 Test to a South African Cohort: a Platform Comparative Prospective Cohort Study. BMC Med. Genet. 14 (1), 20. 10.1186/1471-2350-14-20 23356658 PMC3605304

[B19] Evans W. E. Relling M. V. (1999). Pharmacogenomics: Translating Functional Genomics into Rational Therapeutics. Science 286 (5439), 487–491. 10.1126/science.286.5439.487 10521338

[B20] Freyer N. Greuel S. Knöspel F. Gerstmann F. Storch L. Damm G. (2018). Microscale 3D Liver Bioreactor for *In Vitro* Hepatotoxicity Testing under Perfusion Conditions. Bioengineering 5 (1), 24. 10.3390/bioengineering5010024 PMC5874890 29543727

[B21] Gurdasani D. Carstensen T. Tekola-Ayele F. Pagani L. Tachmazidou I. Hatzikotoulas K. (2015). The African Genome Variation Project Shapes Medical Genetics in Africa. Nature 517 (7534), 327–332. 10.1038/nature13997 25470054 PMC4297536

[B22] Hannan N. R. F. Segeritz C.-P. Touboul T. Vallier L. (2013). Production of Hepatocyte-like Cells from Human Pluripotent Stem Cells. Nat. Protoc. 8 (2), 430–437. 10.1038/nprot.2012.153 23424751 PMC3673228

[B23] Heidariyan Z. Ghanian M. H. Ashjari M. Farzaneh Z. Najarasl M. Rezaei Larijani M. (2018). Efficient and Cost-Effective Generation of Hepatocyte-like Cells through Microparticle-Mediated Delivery of Growth Factors in a 3D Culture of Human Pluripotent Stem Cells. Biomaterials 159, 174–188. 10.1016/j.biomaterials.2018.01.005 29329052

[B24] Hendriks D. F. G. Fredriksson Puigvert L. Messner S. Mortiz W. Ingelman-Sundberg M. (2016). Hepatic 3D Spheroid Models for the Detection and Study of Compounds with Cholestatic Liability. Sci. Rep. 6 (1), 35434. 10.1038/srep35434 27759057 PMC5069690

[B25] Heslop J. A. Rowe C. Walsh J. Sison-Young R. Jenkins R. Kamalian L. (2017). Mechanistic Evaluation of Primary Human Hepatocyte Culture Using Global Proteomic Analysis Reveals a Selective Dedifferentiation Profile. Arch. Toxicol. 91 (1), 439–452. 10.1007/s00204-016-1694-y 27039104 PMC5225178

[B26] Higgins M. J. Stearns V. (2010). CYP2D6 Polymorphisms and Tamoxifen Metabolism: Clinical Relevance. Curr. Oncol. Rep. 12 (1), 7–15. 10.1007/s11912-009-0076-5 20425602

[B27] Hockemeyer D. Jaenisch R. (2016). Induced Pluripotent Stem Cells Meet Genome Editing. Cell Stem Cell 18 (5), 573–586. 10.1016/j.stem.2016.04.013 27152442 PMC4871596

[B28] HPSC registry (2021). HPSC Registry. Available at: https://hpscreg.eu/ (Accessed: October 29, 2021).

[B29] Hurrell T. Kastrinou-Lampou V. Fardellas A. Hendriks D. F. G. Nordling Å. Johansson I. (2020). Human Liver Spheroids as a Model to Study Aetiology and Treatment of Hepatic Fibrosis. Cells 9 (4), 964. 10.3390/cells9040964 PMC7227007 32295224

[B30] Ingelman‐Sundberg M. Lauschke V. M. (2022). 3D Human Liver Spheroids for Translational Pharmacology and Toxicology. Basic Clin. Pharma Tox. 130 (S1), 5–15. 10.1111/bcpt.13587 33872466

[B31] Kamei K.-i. Yoshioka M. Terada S. Tokunaga Y. Chen Y. (2019). Three-dimensional Cultured Liver-On-A-Chip with Mature Hepatocyte-like Cells Derived from Human Pluripotent Stem Cells. Biomed. Microdevices 21 (3), 73. 10.1007/s10544-019-0423-8 31304567

[B32] Kozyra M. Johansson I. Nordling Å. Ullah S. Lauschke V. M. Ingelman-Sundberg M. (2018). Human Hepatic 3D Spheroids as a Model for Steatosis and Insulin Resistance. Sci. Rep. 8 (1), 14297. 10.1038/s41598-018-32722-6 30250238 PMC6155201

[B33] Langmia I. M. (2021). ‘CYP2B6 Functional Variability in Drug Metabolism and Exposure across Populations—Implication for Drug Safety, Dosing, and Individualized Therapy’, Frontiers In Genetics, 12. Available at: https://www.frontiersin.org/article/10.3389/fgene.2021.692234 (Accessed: January 21, 2022). 10.3389/fgene.2021.692234 PMC8313315 34322158

[B34] Lauschke V. M. Shafagh R. Z. Hendriks D. F. G. Ingelman‐Sundberg M. (2019). 3D Primary Hepatocyte Culture Systems for Analyses of Liver Diseases, Drug Metabolism, and Toxicity: Emerging Culture Paradigms and Applications. Biotechnol. J. 14 (7), 1800347. 10.1002/biot.201800347 30957976

[B35] Lauschke V. M. Vorrink S. U. Moro S. M. L. Rezayee F. Nordling Å. Hendriks D. F. G. (2016). Massive Rearrangements of Cellular MicroRNA Signatures Are Key Drivers of Hepatocyte Dedifferentiation. Hepatology 64 (5), 1743–1756. 10.1002/hep.28780 27532775

[B36] LeCluyse E. L. Witek R. P. Andersen M. E. Powers M. J. (2012). Organotypic Liver Culture Models: Meeting Current Challenges in Toxicity Testing. Crit. Rev. Toxicol. 42 (6), 501–548. 10.3109/10408444.2012.682115 22582993 PMC3423873

[B37] Liebler D. C. Guengerich F. P. (2005). Elucidating Mechanisms of Drug-Induced Toxicity. Nat. Rev. Drug Discov. 4 (5), 410–420. 10.1038/nrd1720 15864270

[B38] Masimirembwa C. Hasler J. (2013). Pharmacogenetics in Africa, an Opportunity for Appropriate Drug Dosage Regimens: on the Road to Personalized Healthcare. CPT: Pharmacometrics Syst. Pharmacol. 2 (5), 45. 10.1038/psp.2013.17

[B39] Mathapati S. Siller R. Impellizzeri A. A. Lycke M. Vegheim K. Almaas R. (2016). Small-Molecule-Directed Hepatocyte-like Cell Differentiation of Human Pluripotent Stem Cells. Curr. Protoc. Stem Cel Biol 38 (1), 1G–18G. 10.1002/cpsc.13 27532814

[B40] Mayati A. Moreau A. Le Vée M. Bruyère A. Jouan E. Denizot C. (2018). Functional Polarization of Human Hepatoma HepaRG Cells in Response to Forskolin. Sci. Rep. 8 (1), 16115. 10.1038/s41598-018-34421-8 30382126 PMC6208432

[B41] Moore E. Allen J. B. Mulligan C. J. Wayne E. C. (2021). Ancestry of Cells Must Be Considered in Bioengineering. Nat. Rev. Mater. 7, 2–4. 10.1038/s41578-021-00397-7 PMC10190130 37200939

[B42] Mpye K. L. Matimba A. Dzobo K. Chirikure S. Wonkam A. Dandara C. (2017). Disease burden and the Role of Pharmacogenomics in African Populations. Glob. Health Epidemiol. 2. 10.1017/gheg.2016.21 PMC5870420 29868213

[B43] Nemaura T. Nhachi C. Masimirembwa C. (2012). Impact of Gender, Weight and CYP2B6 Genotype on Efavirenz Exposure in Patients on HIV/AIDS and TB Treatment: Implications for Individualising Therapy. Afr. J. Pharm. Pharmacol. 6 (29), 2188–2193. 10.5897/AJPP12.076

[B44] Ngaimisi E. Mugusi S. Minzi O. Sasi P. Riedel K.-D. Suda A. (2011). Effect of Rifampicin and CYP2B6 Genotype on Long-Term Efavirenz Autoinduction and Plasma Exposure in HIV Patients with or without Tuberculosis. Clin. Pharmacol. Ther. 90 (3), 406–413. 10.1038/clpt.2011.129 21814190

[B45] Ölander M. Wiśniewski J. R. Flörkemeier I. Handin N. Urdzik J. Artursson P. (2019). A Simple Approach for Restoration of Differentiation and Function in Cryopreserved Human Hepatocytes. Arch. Toxicol. 93 (3), 819–829. 10.1007/s00204-018-2375-9 30560367

[B46] Otieno M. A. Gan J. Proctor W. (2018). “Status and Future of 3D Cell Culture in Toxicity Testing,” in Drug-Induced Liver Toxicity. Editors Chen M. Will Y. (New York, NY: Springer New York Methods in Pharmacology and Toxicology), 249–261. 10.1007/978-1-4939-7677-5_12

[B47] Ouchi R. Togo S. Kimura M. Shinozawa T. Koido M. Koike H. (2019). Modeling Steatohepatitis in Humans with Pluripotent Stem Cell-Derived Organoids. Cel Metab. 30 (2), 374–384. e6. 10.1016/j.cmet.2019.05.007 PMC6687537 31155493

[B48] Pingitore P. Sasidharan K. Ekstrand M. Prill S. Lindén D. Romeo S. (2019). Human Multilineage 3D Spheroids as a Model of Liver Steatosis and Fibrosis. Ijms 20 (7), 1629. 10.3390/ijms20071629 PMC6480107 30986904

[B49] Prill S. Caddeo A. Baselli G. Jamialahmadi O. Dongiovanni P. Rametta R. (2019). The TM6SF2 E167K Genetic Variant Induces Lipid Biosynthesis and Reduces Apolipoprotein B Secretion in Human Hepatic 3D Spheroids. Sci. Rep. 9 (1), 11585. 10.1038/s41598-019-47737-w 31406127 PMC6690969

[B50] Qiu G.-H. Xie X. Xu F. Shi X. Wang Y. Deng L. (2015). Distinctive Pharmacological Differences between Liver Cancer Cell Lines HepG2 and Hep3B. Cytotechnology 67 (1), 1–12. 10.1007/s10616-014-9761-9 25002206 PMC4294832

[B51] Radouani F. Zass L. Hamdi Y. Rocha J. D. Sallam R. Abdelhak S. (2020). A Review of Clinical Pharmacogenetics Studies in African Populations. Personalized Med. 17 (2), 155–170. 10.2217/pme-2019-0110 PMC8093600 32125935

[B52] Raggi C. M'Callum M. A. Pham Q. T. Gaub P. Selleri S. Baratang N. V. (2022). Leveraging Interacting Signaling Pathways to Robustly Improve the Quality and Yield of Human Pluripotent Stem Cell-derived Hepatoblasts and Hepatocytes. Stem Cell Rep. 17 (3), 584–598. 10.1016/j.stemcr.2022.01.003 PMC9039749 35120625

[B53] Rajman I. Knapp L. Morgan T. Masimirembwa C. (2017). African Genetic Diversity: Implications for Cytochrome P450-Mediated Drug Metabolism and Drug Development. EBioMedicine 17, 67–74. 10.1016/j.ebiom.2017.02.017 28237373 PMC5360579

[B54] Ramboer E. De Craene B. De Kock J. Vanhaecke T. Berx G. Rogiers V. (2014). Strategies for Immortalization of Primary Hepatocytes. J. Hepatol. 61 (4), 925–943. 10.1016/j.jhep.2014.05.046 24911463 PMC4169710

[B55] Ramboer E. Vanhaecke T. Rogiers V. Vinken M. (2015). Immortalized Human Hepatic Cell Lines for *In Vitro* Testing and Research Purposes. Methods Mol. Biol. (Clifton, N.J.) 1250, 53–76. 10.1007/978-1-4939-2074-7_4 PMC4579543 26272134

[B56] Rashidi H. Luu N.-T. Alwahsh S. M. Ginai M. Alhaque S. Dong H. (2018). 3D Human Liver Tissue from Pluripotent Stem Cells Displays Stable Phenotype *In Vitro* and Supports Compromised Liver Function *In Vivo* . Arch. Toxicol. 92 (10), 3117–3129. 10.1007/s00204-018-2280-2 30155720 PMC6132688

[B57] Sampaziotis F. Segeritz C. P. Vallier L. (2015). Potential of Human Induced Pluripotent Stem Cells in Studies of Liver Disease. Hepatology 62 (1), 303–311. 10.1002/hep.27651 25502113

[B58] Sanjurjo-Soriano C. (2021). “CRISPR/Cas9-Mediated Genome Editing to Generate Clonal iPSC Lines,” in Methods in Molecular Biology. Editors Erkilic N. Mamaeva D. Kalatzis V.. [Preprint]. 10.1007/7651_2021_362 33755901

[B59] Schärfe C. P. I. Tremmel R. Schwab M. Kohlbacher O. Marks D. S. (2017). Genetic Variation in Human Drug-Related Genes. Genome Med. 9 (1), 117. 10.1186/s13073-017-0502-5 29273096 PMC5740940

[B60] Segeritz C.-P. Rashid S. T. de Brito M. C. Serra M. P. Ordonez A. Morell C. M. (2018). hiPSC Hepatocyte Model Demonstrates the Role of Unfolded Protein Response and Inflammatory Networks in α1-antitrypsin Deficiency. J. Hepatol. 69 (4), 851–860. 10.1016/j.jhep.2018.05.028 29879455 PMC6562205

[B61] Shrif N. E. M. A. Won H.-H. Lee S.-T. Park J.-H. Kim K.-K. Kim M.-J. (2011). Evaluation of the Effects of VKORC1 Polymorphisms and Haplotypes, CYP2C9 Genotypes, and Clinical Factors on Warfarin Response in Sudanese Patients. Eur. J. Clin. Pharmacol. 67 (11), 1119–1130. 10.1007/s00228-011-1060-1 21590310

[B62] Si-Tayeb K. Noto F. K. Nagaoka M. Li J. Battle M. A. Duris C. (2010). Highly Efficient Generation of Human Hepatocyte-like Cells from Induced Pluripotent Stem Cells. Hepatology 51 (1), 297–305. 10.1002/hep.23354 19998274 PMC2946078

[B63] Stark Z. Dolman L. Manolio T. A. Ozenberger B. Hill S. L. Caulfied M. J. (2019). Integrating Genomics into Healthcare: A Global Responsibility. Am. J. Hum. Genet. 104 (1), 13–20. 10.1016/j.ajhg.2018.11.014 30609404 PMC6323624

[B64] Szabo M. Veres Z. Baranyai Z. Jakab F. Jemnitz K. (2013). Comparison of Human Hepatoma HepaRG Cells with Human and Rat Hepatocytes in Uptake Transport Assays in Order to Predict a Risk of Drug Induced Hepatotoxicity. PLOS ONE 8 (3), e59432. 10.1371/journal.pone.0059432 23516635 PMC3597610

[B65] Takahashi K. Tanabe K. Ohnuki M. Narita M. Ichisaka T. Tomoda K. (2007). Induction of Pluripotent Stem Cells from Adult Human Fibroblasts by Defined Factors. Cell 131 (5), 861–872. 10.1016/j.cell.2007.11.019 18035408

[B66] Takebe T. Sekine K. Kimura M. Yoshizawa E. Ayano S. Koido M. (2017). Massive and Reproducible Production of Liver Buds Entirely from Human Pluripotent Stem Cells. Cel Rep. 21 (10), 2661–2670. 10.1016/j.celrep.2017.11.005 29212014

[B67] Takeishi K. Collin de l’Hortet A. Wang Y. Handa K. Guzman-Lepe J. Matsubara K. (2020). Assembly and Function of a Bioengineered Human Liver for Transplantation Generated Solely from Induced Pluripotent Stem Cells. Cel Rep. 31 (9), 107711. 10.1016/j.celrep.2020.107711 PMC7734598 32492423

[B68] Touboul T. Hannan N. R. F. Corbineau S. Martinez A. Martinet C. Branchereau S. (2010). Generation of Functional Hepatocytes from Human Embryonic Stem Cells under Chemically Defined Conditions that Recapitulate Liver Development. Hepatology 51 (5), 1754–1765. 10.1002/hep.23506 20301097

[B69] van der Merwe N. Bouwens C. S. H. Pienaar R. van der Merwe L. Yako Y. Y. Geiger D. H. (2012). CYP2D6 Genotyping and Use of Antidepressants in Breast Cancer Patients: Test Development for Clinical Application. Metab. Brain Dis. 27 (3), 319–326. 10.1007/s11011-012-9312-z 22638694 PMC3505529

[B70] Vorrink S. U. Ullah S. Schmidt S. Nandania J. Velagapudi V. Beck O. (2017). Endogenous and Xenobiotic Metabolic Stability of Primary Human Hepatocytes in Long‐term 3D Spheroid Cultures Revealed by a Combination of Targeted and Untargeted Metabolomics. FASEB j. 31 (6), 2696–2708. 10.1096/fj.201601375R 28264975 PMC5434660

[B71] Vorrink S. U. Zhou Y. Ingelman-Sundberg M. Lauschke V. M. (2018). Prediction of Drug-Induced Hepatotoxicity Using Long-Term Stable Primary Hepatic 3D Spheroid Cultures in Chemically Defined Conditions. Toxicol. Sci. 163 (2), 655–665. 10.1093/toxsci/kfy058 29590495 PMC5974779

[B72] Weinshilboum R. (2003). Inheritance and Drug Response. N. Engl. J. Med. 348 (6), 529–537. 10.1056/NEJMra020021 12571261

[B73] Wong N. (2000). A Comprehensive Karyotypic Study on Human Hepatocellular Carcinoma by Spectral Karyotyping. Hepatology 32 (5), 1060–1068. 10.1053/jhep.2000.19349 11050057

[B74] Wonkam A. (2021). Sequence Three Million Genomes across Africa. Nature 590 (7845), 209–211. 10.1038/d41586-021-00313-7 33568829 PMC9979155

[B75] Yamasaki C. Ishida Y. Yanagi A. Yoshizane Y. Kojima Y. Ogawa Y. (2020). Culture Density Contributes to Hepatic Functions of Fresh Human Hepatocytes Isolated from Chimeric Mice with Humanized Livers: Novel, Long-Term, Functional Two-Dimensional *In Vitro* Tool for Developing New Drugs. PLOS ONE 15 (9), e0237809. 10.1371/journal.pone.0237809 32915792 PMC7485858

[B76] Yusa K. Rashid S. T. Strick-Marchand H. Varela I. Liu P.-Q. Paschon D. E. (2011). Targeted Gene Correction of α1-antitrypsin Deficiency in Induced Pluripotent Stem Cells. Nature 478 (7369), 391–394. 10.1038/nature10424 21993621 PMC3198846

[B77] Zabulica M. Jakobsson T. Ravaioli F. Vosough M. Gramignoli R. Ellis E. (2021). Gene Editing Correction of a Urea Cycle Defect in Organoid Stem Cell Derived Hepatocyte-like Cells. Ijms 22 (3), 1217. 10.3390/ijms22031217 33530582 PMC7865883

[B78] Zabulica M. Srinivasan R. C. Vosough M. Hammarstedt C. Wu T. Gramignoli R. (2019). Guide to the Assessment of Mature Liver Gene Expression in Stem Cell-Derived Hepatocytes. Stem Cell Dev. 28 (14), 907–919. 10.1089/scd.2019.0064 PMC6648222 31122128

[B79] Zanger U. M. Schwab M. (2013). Cytochrome P450 Enzymes in Drug Metabolism: Regulation of Gene Expression, Enzyme Activities, and Impact of Genetic Variation. Pharmacol. Ther. 138 (1), 103–141. 10.1016/j.pharmthera.2012.12.007 23333322

[B80] Zeilinger K. Freyer N. Damm G. Seehofer D. Knöspel F. (2016). Cell Sources for *In Vitro* Human Liver Cell Culture Models. Exp. Biol. Med. (Maywood) 241 (15), 1684–1698. 10.1177/1535370216657448 27385595 PMC4999620

[B81] Zhang D. Surapaneni S. (2012). “Appendix: Drug Metabolizing Enzymes and Biotransformation Reactions,” in ADME-enabling Technologies in Drug Design and Development. Editors Penner N. Woodward C. Prakash C. (Hoboken, NJ, USA: John Wiley & Sons), 545–565. 10.1002/9781118180778.app1

[B82] Zhou D. Fan J.-G. (2019). Microbial Metabolites in Non-alcoholic Fatty Liver Disease. Wjg 25 (17), 2019–2028. 10.3748/wjg.v25.i17.2019 31114130 PMC6506577

